# Methyl-Cantharidimide Inhibits Growth of Human Hepatocellular Carcinoma Cells by Inducing Cell Cycle Arrest and Promoting Apoptosis

**DOI:** 10.3389/fonc.2019.01234

**Published:** 2019-11-15

**Authors:** Xiangzhong Huang, Wen Xie, Xiaofan Yu, Caiyun Fan, Jin Wang, Yi Cao, Jianxiang Li

**Affiliations:** ^1^Department of Interventional Therapy, Affiliated Jiangyin Hospital, Medical College of Southeast University, Jiangyin, China; ^2^School of Public Health, Medical College of Soochow University, Suzhou, China

**Keywords:** methyl-cantharidimide (MCA), cantharidin (CTD), hepatocellular carcinoma, cell growth, invasion

## Abstract

Methyl-Cantharidimide (MCA) is a derivative of cantharidin which has potential anticancer activity. This study investigates the effect of MCA on the growth and metastasis of human hepatocellular carcinoma (HCC) cells. Human HCC HepG2 and Hep3B2.1-7 cells, and normal hepatocytes (L02) were treated with a series of concentrations of MCA. The inhibition ability of these cells was examined by CCK-8 assay. Cell cycle and cell apoptosis were determined using Flow Cytometry. The effect of MCA on cell migration and invasion was evaluated through scratch wound healing and transwell migration assays. Furthermore, Western blot was used to evaluate biomarkers associated with cell cycle and apoptosis. It was found that: (i) MCA inhibited cell proliferation in HCC cells in a dose- and time-dependent manner, especially in HepG2 cells; (ii) MCA arrested HCC cells in G-1 phase cell cycle; (iii) MCA induced HCC cells apoptosis; (iv) MCA inhibited the migration ability of HCC cells; and (v) MCA treatment significantly increased cleaved-caspase3 and decreased NF-κB protein in HCC cells. These results suggest that MCA has cytotoxic effect on HCC cells by inducing cell cycle arrest and promoting apoptosis. MCA could be developed as an previous anticancer drug for the treatment of human hepatocellular carcinoma.

## Introduction

Hepatocellular carcinoma (HCC) is a primary malignant tumor in the liver. It is the third leading cause of cancer deaths worldwide. The annual incidence of HCC in North America and Western Europe ranges from 10 to 15 cases per 100,000 people, while in parts of Africa and Asia, the incidence is much higher and range from 50 to 150 cases per 100,000 people (1). The median survival following diagnosis ranges from ~6 to 20 months. Currently, tumor resection is an option of choice for the treatment of HCC. However, since most of the diagnosis is made in late stages of the disease, HCC patients have few opportunity to undergo surgery. Thus, there is an urgent need for early diagnosis and effective therapeutic medication (2, 3). In recent years, there were several reports suggest that some traditional Chinese medicines showed remarkable anti-cancer activity (4–7). Cantharidin (CTD) is a biologically active ingredient isolated from Chinese blister Beetle, *Mylabris phalerata*. It is used in traditional Chinese medicine for more than 2000 years to treat various diseases including cancers (8–14). Several recent reports have identified potential mechanisms of action of cantharidin in different types of human cancer cells, including hepatocellular carcinoma cells (15–21). Although CTD is a natural toxin possessing potent anti-tumor properties, its use in cancer treatment has been limited because of severe side effects due to its highly toxic nature. Analogs of chemically modified CTD have been synthesized to achieve a comparable anti-tumor property with less toxic effect (22).

Methyl-cantharidimide (MCA, 2,3a,7a-Trimethyl-hexahydro-4,7-epoxido-isoindol-1,3-dion, C11H15NO3, molecular weight 209.242) is one such derivative of CTD. MCA is chemically synthesized and developed as a powerful anticancer drug with less toxicity (23). However, the anticancer effect of MCA and its mechanism of action has not been evaluated. This study investigates the anticancer effect of MCA using hepatocellular carcinoma cell models. Human hepatocellular carcinoma cell lines, HepG2 and Hep3B2.1-7 and normal hepatocyte LO_2_ cells were used. Various concentrations of MCA were tested in these cells to determine its tumor growth inhibitory effect. The mechanism of anticancer activity of MCA was explored by determining the change of cell cycle, cell apoptosis and cell migration ability. The activity of key-players that regulate cell apoptosis, caspase-3 and Nuclear Factor kappa-light-chain-enhancer of activated B cells (NF-κB), were evaluated.

## Materials and Methods

### Cell Culture

Human hepatocellular carcinoma cell lines, HepG2 and Hep3B2.1-7, were purchased from Chinese Type Culture Collection, Chinese Academy of Sciences, Shanghai, China. Normal human hepatic cell L02, was a gift from the Department of Toxicology, School of Public Health, Soochow University, Suzhou, China. Cells were cultured in complete Dulbecco's modified Eagle's medium (DMEM) containing 10% fetal bovine serum, 100 μg/ml streptomycin and penicillin (all obtained from Hyclone, Suzhou, China) in an incubator maintaining at 37 ± 0.5°C in a humidified atmosphere with 5% CO_2_. Cells were trypsinized after reaching confluence and aliquots were kept frozen at −80°C. Cells were sub-cultured and those in 3–4 passages were used in experiments described below. All experiments were repeated three times.

### Methyl-Cantharidimide (MCA)

Methyl-Cantharidimide (MCA) was a gift of Sihuan Bioengineering Co., Ltd., Jiangsu, China. A stock solution (20 mM) was freshly prepared by dissolving MCA in dimethyl sulfoxide (DMSO, PanReac Applichem, Germany) and diluted in desired concentrations in complete DMEM. HepG2, Hep3B2.1-7, and LO_2_ cells were treated *in vitro* with a series of final concentrations of MCA or with the solvent DMEM as control.

### Cytotoxicity Essay (IC50)

Two-hundred μl aliquots of HepG2, Hep3B2.1-7 and L02 cells in DMEM complete medium (~3000 cells each) were distributed into 96-well plate and cultured for 24 h at 37 ± 0.5°C. Then, 200 μl MCA solution was added to give a final concentration of 50, 100, 200, 400, and 800 μM. The cells were cultured for 24, 48, and 72 h. The proliferation ability of the cells in each well was assessed using a CCK-8 assay kit (Dojindo, China) according to manufacturer's instructions. Briefly, 20 μl of CCK-8 solution was added to each well and the cells were incubated for 4 h at 37 ± 0.5°C. The plates were then read in the standard plate reader (FilterMax F5, Molecular Devices, USA) at a reference wavelength of 450 nm. The percent inhibition of growth in cells treated with MCA was calculated as follows: % Inhibition = [A450(drug) – A450(blank)]/[A450(control) – A450(blank)] × 100%. The IC30 that was obtained for HepG2 cells was 137.56 μM MCA. This dose was used in subsequent experiments.

### Cell Cycle Evaluation

Two-hundred μl aliquots of HepG2 and Hep3B2.1-7 cells in complete DMEM medium (~1 × 10^5^ cells each) were distributed in 6-well plates and cultured for 24 h at 37 ±−0.5°C. Then, the cells were treated with 137.56 μM MCA (IC30 concentration obtained for HepG2 cells) for 48 h, collected by trypsinization, washed twice with cold phosphate buffered saline (PBS), suspended in cold 70% methanol and left at −20°C overnight. The cells were then washed twice with cold PBS and stained with PBS solution containing 20 μg/ml PI and 50 μg/ml of RNaseA for 30 min. The cell cycle analysis was carried out using a flow cytometer (Beckman coulter, Shanghai, China) (24).

### Cell Apoptosis Detection

Annexin V-FITC apoptosis detection kit (KeyGEN Biotech, Shanghai, China) was used to evaluate cell apoptosis. Two-hundred μl aliquots of HepG2 and Hep3B2.1-7 in complete DMEM medium (~1 × 10^5^ cells each) were distributed in 6-well plates and cultured for 24 h. Then, the cells were treated with 137.56 μM MCA (IC30 concentration obtained for HepG2 cells) for 48 h. The cells were collected by trypsinization, incubated with Annexin V in a buffer containing propidium iodide for 15 min. The percent cells in apoptosis were then determined using a flow cytometer (Beckman coulter, Shanghai, China) (25).

### Scratch Wound Healing Assay

Two hundred microliters aliquots of HepG2 and Hep3B2.1-7 cells in complete DMEM medium (~2 × 10^5^ cells each) were distributed in 6-well plates and cultured for 24 h at 37°C. Then, the cells were treated with 137.56 μM MCA (IC30 concentration obtained for HepG2 cells) for 48 h. Cells were allowed to grow up to 100% confluence and a scratch was made in the plate using with a P10 pipette tip. The cells were cultured in fresh serum-free DMEM medium. images were collected at 0 and 24 h under an inverted microscope (Olympus, Germany) and quantitatively analyzed using the NIH Image J software.

### Transwell Migration Assay

HepG2 and Hep3B2.1-7 cancer cells and MCA treated cells (2 × 10^5^) were seeded in the upper chambers (pore size, 8 μm) of the 6-well plate (Corning, USA) in 1 ml serum-free medium. The lower chambers were filled with 2 ml complete medium with 10% FBS, and the plate was incubated under standard conditions for 24 h. After removing the cells in the upper surface of the membrane with a cotton swab, cells in the lower chamber were fixed with methanol and stained with 0.5% crystal violet solution. The images were taken using an inverted microscope (Olympus, Germany and analyzed using NIH Image J software.

### Western Blot Analysis

Approximated 2 × 10^5^ HepG2 cells were treated with 137.56 μM MCA (IC30 concentration obtained for HepG2 cells) for 48 h. Protein extracts were prepared by lysing the cells in lysis buffer containing 50 mM Tris (pH 7.4), 150 mM sodium chloride, 1% Triton X-100, 1% sodium deoxycholate, 0.1% sodium dodecyl sulfate and 1 mM phenyl-methyl-sulfonyl fluoride (all obtained from Beyotime, Shanghai, China). The cell lysates were centrifuged at 14,000 × *g* for 5 min at 4°C and the supernatant containing solubilized proteins was collected. The protein concentration in all samples was determined by using the BCA protein assay kit (Beyotime, Shanghai, China). From each sample, equal amount of protein (40 μg per lane) was loaded, separated by 10% sodium dodecyl sulfate polyacrylamide gel (SDS–PAGE) and then transferred to polyvinylidene difluoride (PVDF) membranes (Millipore Corporation, Billerica, MA, USA). The membranes were blocked for 2 h in 5% fat-free dry milk (Yili Industrial, Inner Mongolia, China) containing Tween 20-Tris-buffered saline (TTBS). The membranes were then incubated with primary antibodies (rabbit monoclonal anti-NF-κB antibody, rabbit monoclonal anti-cleaved caspase 3 antibody and rabbit monoclonal anti- GADPH, Abcam, Cambridge, USA) overnight at 4°C. They were washed three times in TTBS and incubated further with horseradish peroxidase-conjugated antibodies (Beyotime, Shanghai, China) for 1.5 h at room temperature. This was followed by washing the membranes three times with TTBS. The immunoreactive proteins on the membranes were detected using enhanced chemiluminescence reagents (Millipore Corporation) and G-BOX Chemi XRQ (Syngene, UK). The blots were quantified and normalized with the level of GADPH to correct the differences in loading of the proteins in MCA-treated cells.

### Statistical Analysis

The results from three independent experiments were pooled and analyzed using GraphPad Prism 5.0 (GraphPad Software, San Diego, CA, USA). The data were presented as mean ± SD. Statistical significances of differences were analyzed by one way analysis of variance (ANOVA) test followed by Sidak's multiple comparison post-test. A *p*-value of <0.05 was considered as significant difference between MCA treated and untreated cells.

## Results

### MCA Inhibits the Proliferation of HCC Cells With Little Effect on the Proliferation of Normal Hepatic Cells

The effect of MCA on the proliferation of HCC and normal hepatic cells was evaluated using CCK 8 assay. The inhibition rate of growth in L02, HepG2 and Hep3B2.1-7 cells treated with 50, 100, 200, 400 and, 800 μM MCA were presented. In all three types of cells, there was a decrease in growth which was positively correlated with not only the increased concentration of MCA but also the duration of treatment from 24 to 72 h. The IC50 observed in HepG2 and Hep3B2.17 HCC cells and normal LO_2_ hepatic cells treated with MCA for 24, 48, and 72 h were presented in Table 1. The IC50 observed in LO_2_, HepG2 and Hep3B2.1-7 cells, after 48 h treatment with MCA were at concentrations of 363.56 ± 44.46, 227.00 ± 6.11, and 273.44 ± 18.52 μM, respectively. This result indicated that MCA inhibits the proliferation of HepG2 and Hep3B2.1-7 cells with little effect on the proliferation of normal hepatocyte LO_2_ cells.

**Table 1:** IC50 of MCA in HCC cells and normal hepatic cells.

**Cell type**	**Treatment duration**		
	**24 h**	**48 h**	**72 h**
HepG2	301.49 ± 21.48^*^	227.00 ± 6.11^*^^#^	157.00 ± 3.98^*^^#^
Hep3B2.1-7	295.63 ± 25.61^*^	273.44 ± 18.52^*^	238.10 ± 7.43^*^^#^
L02	583.92 ± 63.17	363.56 ± 44.46^#^	347.40 ± 31.53^#^

*All experiments were repeated three times. Data are mean ± Standard Deviation*.

* p < 0.05: Compared to L02 cells;

# *p < 0.05: Compared to 24 h*.

### MCA Induces Cell Cycle Arrest at G1/S of HCC Cells

**Figure 1:**
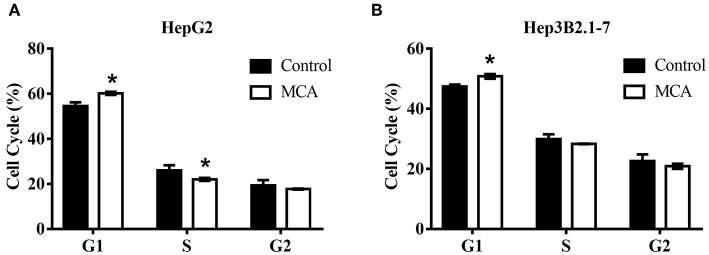
Flow cytometry on cell cycle analysis in HepG2 and Hep3B2.1-7 cells following treatment with MCA for 48 h. **(A)** Effect of MCA on cell cycle of HepG2 cells. **(B)** Effect of MCA on cell cycle of Hep3B2.1-7 cells. *p <0.05 via control.

Since MCA inhibits HCC cell proliferation, we next examine whether MCA affects cell cycle progression in HCC cells by flow cytometry. HepG2 and Hep3B2.1-7 cells were treated with and without MCA for 48 hrs. The IC30 concentration of MCA obtained from HepG2 cells in the above experiment 137.56 μM was used. It was found that MCA treatment blocks the progression of cell cycle from G1- to S-phase in both HepG2 and Hep3B2.1-7 HCC cells, resulting in increased cell number in G1-phase. The percent of HepG2 cells at G1-phase was 60.23 ± 0.70 which was significantly higher than in solvent-treated cells, which was 54.58 ± 1.67 (*p* < 0.05). Similarly, the percent of Hep3B2.1-7 cells at G1-phase was 50.81 ± 0.76, which was significantly higher than that in solvent-treated cells, 47.48 ± 0.62 (*p* < 0.05). There was no significant difference in percent cells in S- and G2-phase in both cell types (*p* > 0.05). This result indicates that MCA induces cell cycle arrest at G1/S phase (Table 2 and Figures 1A,B).

**Table 2:** Alteration of cell cycle in HepG2 and Hep3B2.1-7 HCC cells treated with MCA.

**Cell type**	**Treatment**	**G1**	**S**	**G2**
HeoG2	Solvent	54.58 ± 1.67	26.02 ± 2.27	19.39 ± 2.33
	MCA	60.23 ± 0.70^*^	22.04 ± 0.65^*^	17.73 ± 0.22
Hep3B2.1-7	Solvent	47.48 ± 0.62	29.92 ± 1.62	22.60 ± 2.22
	MCA	50.81 ± 0.76^*^	28.31 ± 0.09	20.88 ± 0.83

*All experiments were repeated three times. MCA treatment with IC30 concentration (137.56 μM) for 48 h. Data are mean ± Standard Deviation*.

* *p < 0.05*.

### MCA Promotes Apoptosis of HCC Cells

**Figure 2:**
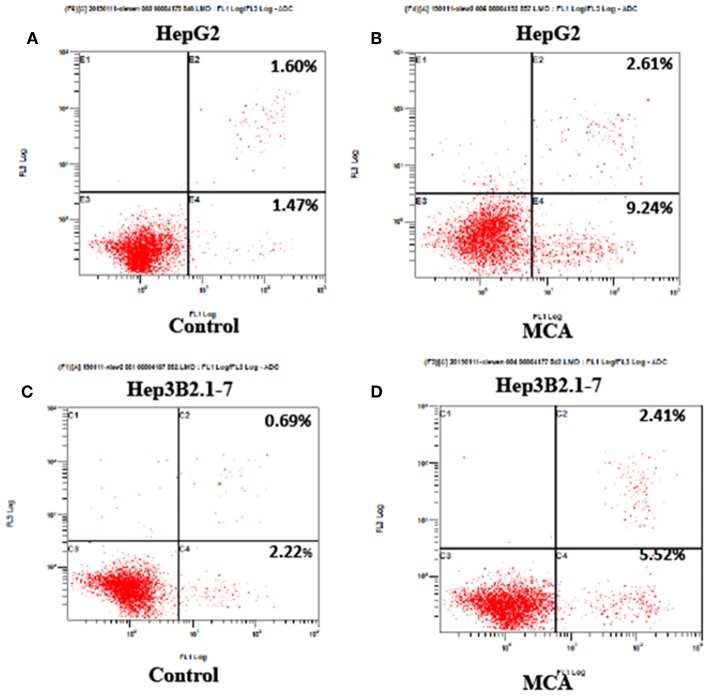
Flow cytometry on cell apoptosis analysis in HepG2 and Hep3B2.1-7 cells following treatment with MCA for 48 h. **(A)** Apoptosis of untreated HepG2 cells for 48 h. **(B)** Apoptosis of HepG2 cell treated with MCA for 48 h. **(C)** Apoptosis of untreated Hep3B2.1-7 cells for 48 h. **(D)** Apoptosis of Hep3B2.1-7 cell treated with MCA for 48 h.

The effect of MCA on cell apoptosis of HCC cells was evaluated by flow cytometry. The early, late and total cell apoptosis in HepG2 and Hep3B2.1-7 cells, treated with or without 137.56 μM MCA for 48 h were presented in Table 3 and Figure 2. The data showed a significant increase in total percent of cells of apoptosis in both HepG2 and Hep3B2.1-7 cells: 12.34 ± 2.81 and 7.49 ± 0.42% in HepG2 and Hep3B2.1-7 cells, respectively compared with solvent-treated cells, 2.83 ± 1.04 and 3.70 ± 1.48%, respectively. This result indicates that MCA treatment promotes apoptosis of HCC cells.

**Table 3:** Apoptosis of HepG2 and Hep3B2.1-7 cells after treatment with MCA (mM).

**Cell type**	**Treatment**	**Early apoptosis**	**Late apoptosis**	**Total apoptosis**
HepG2	Solvent	1.29 ± 0.42	1.53 ± 0.62	2.83 ± 1.04
	MCA	9.26 ± 2.27^*^	3.08 ± 0.65^*^	12.34 ± 2.81^*^
Hep3B2.1-7	Solvent	2.56 ± 0.88	1.14 ± 0.62	3.70 ± 1.48
	MCA	5.44 ± 0.38^*^	2.04 ± 0.38	7.49 ± 0.42^*^

*All experiments were repeated three times. MCA treatment with IC30 concentration (137.56 μM) for 48 h. Data are mean ± Standard Deviation*.

* *p < 0.05*.

### MCA Inhibits Cell Migration

**Figure 3:**
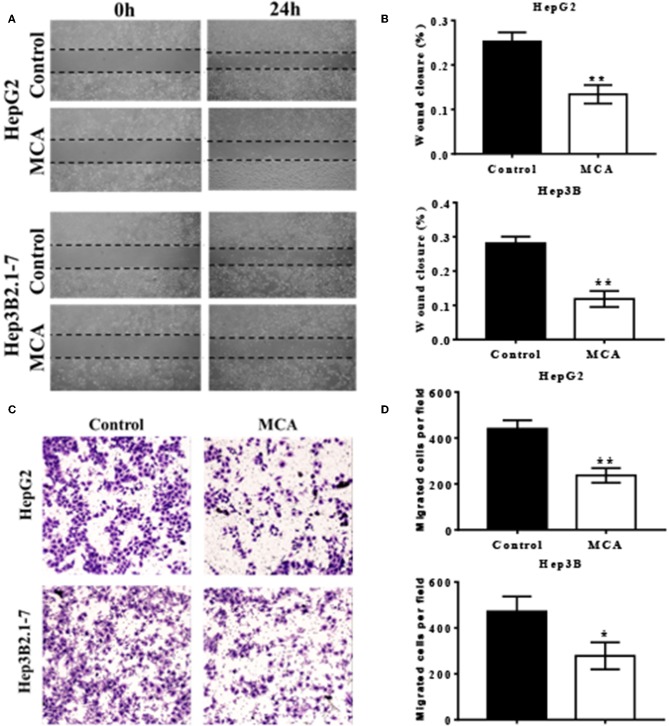
Scratch wound healing assays performed in HepG2 and Hep3B2.1-7 cells treated with conditioned medium containing MCA or vehicle control for 48 h **(A)**. Quantitative analysis of wound closure **(B)**. HepG2 and Hep3B2.1-7 cells were treated with the indicated conditioned medium containing MCA or vehicle control for 48 h in a transwell assay **(C)**. Cells that migrated through transwells were stained with crystal violet and photomicrographed. Quantitative analysis of migrated cells perfield **(D)**. *p < 0.05, **p < 0.01 via Control.

To investigate the effect of MCA in the migratory ability of HCC cells, we performed scratch wound healing and transwell cell migration assays. Scratch wound healing assay indicated that MCA significantly inhibited the migration ability of HepG2 and Hep3B2.1-7 cells (Figures 3A,B). As shown in Figures 3C,D, further transwell migration assay demonstrated that the migrated cells were significantly decreased in MCA treated HepG2 and Hep3B2.1-7 cells compared to the untreated cells.

### MCA Up-Regulates the Expression of Cleaved Caspase-3 and Down-Regulates the Expression of NF-κB Proteins

**Figure 4:**
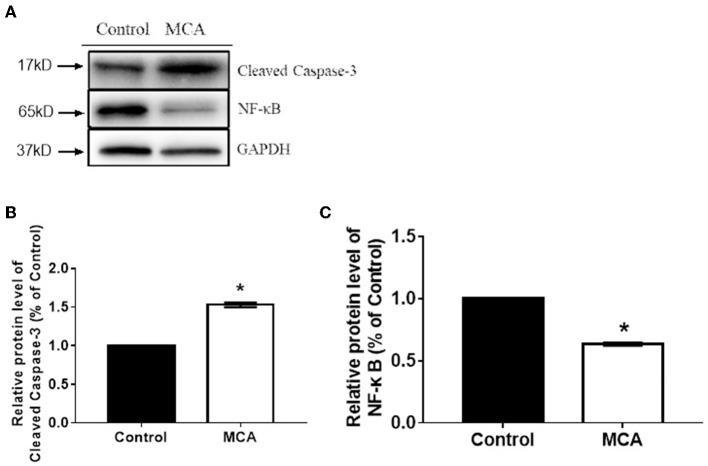
Western blot analysis on the expression of cleaved caspase-3 and NF-κB protein in HCC cells (HepG2 and Hep3B2.1-7) following treatment with MCA for 48 h. **(A)** the bar graph illustrates data normalized to house-keeping genes (GAPDH) and expressed as % control. Cleaved-Caspase3 and NF-κB protein expression levels in the control and MCA groups. **(B)** The bar graph of Cleaved-Caspase3 proteins extracted from HepG2 cell. **(C)** The Bar graph of BF-κB protein extracted from HepG2 cell. *p < 0.05 via Control.

Because MCA induces apoptosis of HCC cells, we explored whether the molecules that regulate cell apoptosis were involved. Western blot analysis on the protein expression of cleaved caspase-3 and NF-κB was performed and the results were presented in Figure 4. The data showed significantly increased level of caspase-3 protein (*p* < 0.05) and significantly decreased level of NF-κB protein (*p* < 0.05) in HepG2 cells treated with 137.56 μM MCA for 48 hrs. This result suggested that MCA up-regulates the expression of Cleaved Caspase-3 and down-regulates the expression of NF-κB proteins.

## Discussion

Hepatocellular carcinoma is the primary malignant tumor in liver. It is usually associated with liver cirrhosis, viral hepatitis, aflatoxin exposure, and alcohol abuse. Typically, HCC is diagnosed in late stages and thus, patients have few opportunity for surgical resection or transplantation (2, 3). Currently, there is no ideal treatment option for the cure of the disease. In recent years, there have been increasing efforts to develop drugs for the treatment and prevention of HCC. Cantharidin (CTD) is a biologically active ingredient isolated from the Chinese blister beetle (*Mylabris phalerata*) and has been used in traditional Chinese medicine for over 2000 years to treat various diseases including cancers (8–14). However, the dose of CTD used for cancer treatment is very close to its toxic dose and its use had been hampered in clinics. Nonetheless, researchers have identified the pharmacological and toxicological properties of CTD (26–30), and started to synthesize and develop bioactive derivatives that have anti-cancer potential with low toxicological profile (31).

MCA is one of such synthetic derivatives of CTD (23). Its anti-cancer effect and the mechanism of action particularly in hepatocellular carcinoma cells has not been delineated. In this study, MCA was tested in a series of concentrations to determine its growth inhibitory effect in different human HCC cells, HepG2, and Hep3B2.1-7 cells, and compared with the normal LO_2_ hepatic cells. The 50% growth inhibitory effect of MCA in HepG2 and Hep3B2.1-7 cells was observed at a concentration of 226.82 and 273.18 μM, respectively, while that in normal hepatic LO_2_ cells was at 379.41 μM. This result suggests that normal hepatic cells required a higher concentration of MCA to elicit 50% growth inhibitory effect. In other words, MCA was selectively toxic to HCC cells compared with that in normal hepatic cells. We also found that treatment of HepG2 and Hep3B2.1-7 HCC cells with IC30 dose of MCA induces cell cycle G1/S arrest, resulting in accumulation of cells in G1 phase of the cell cycle. Flow cytometric analysis showed that the IC30 dose of MCA induced significantly higher percent of cell apoptosis in HepG2 and Hep3B2.1-7 cells compared with normal hepatic cells. These observations suggested that MCA exert growth inhibitory effect and induce cell apoptosis in HCC cells. Transwell migration assay demonstrated that the migrated cells were significantly decreased in MCA treated HepG2 and Hep3B2.1-7 cells compared to the untreated cells. Caspases are crucial mediators of cell apoptosis, among them, caspase-3 is a frequently activated protease, catalyzing the specific cleavage of key cellular proteins. Caspase-3 is indispensable for apoptotic chromatin condensation and DNA fragmentation. Thus, caspase-3 is essential for certain processes associated with the formation of apoptotic bodies, but it may also function before or at the stage when commitment to loss of cell viability is made (32, 33). NF-κB is a primary transcription factor which plays a key role in several cellular processes including cell proliferation and survival (34–36). In this study, 48 h after treatment of HepG2 cells with MCA, the protein level of cleaved-caspase3 was significantly increased while that of NF-κB was significantly decreased. These results suggested that both cleaved caspase-3 and NF-κB played important roles in MCA-induced cell apoptosis in HCC cells. Our results are in consistent with other study which showed that MCA can inhibit the proliferation of HCC cells and prolong the survival of mice with hepatocellular carcinoma (37). Studies on sodium cantharidate, another derivative of cantharidin, have also shown that sodium cantharidate can inhibit the growth of liver cancer cells and induce cell apoptosis (38). Therefore, the inhibitory effect of CTD derivatives on HCC cells is most likely associated with inhibiting cancer cell growth and inducing cancer cell apoptosis by activating caspace-3 and inhibiting NF-κB. The mechanism of action of MCA *in vivo* warrants further investigation.

## Conclusions

Our results suggest that MCA can inhibit cell proliferation and migration of cancer cells. This study provides a rational for developing MCA as a therapeutic agent for the treatment of hepatocellular carcinoma.

## Data Availability Statement

Data will be available upon request by writing to the corresponding author.

## Author Contributions

XH performed MTT assay and write the first draft. XH, WX, CF, and XY performed flow cytometry, cell migration, and invasion assays. CF and JW performed Western blotting, YC performed statistics. JL conceived the study, supervise experiments and results analysis. All authors edited the manuscript and approved the final version.

### Conflict of Interest

The authors declare that the research was conducted in the absence of any commercial or financial relationships that could be construed as a potential conflict of interest.
